# How many sexual minorities are hidden? Projecting the size of the global closet with implications for policy and public health

**DOI:** 10.1371/journal.pone.0218084

**Published:** 2019-06-13

**Authors:** John E. Pachankis, Richard Bränström

**Affiliations:** 1 Department of Social and Behavioral Sciences, Yale School of Public Health, New Haven, CT, United States of America; 2 Department of Clinical Neuroscience, Karolinska Institutet, Stockholm, Sweden; University of California Los Angeles, UNITED STATES

## Abstract

Because sexual orientation concealment can exact deep mental and physical health costs and dampen the public visibility necessary for advancing equal rights, estimating the proportion of the global sexual minority population that conceals its sexual orientation represents a matter of public health and policy concern. Yet a historic lack of cross-national datasets of sexual minorities has precluded accurate estimates of the size of the global closet. We extrapolated the size of the global closet (i.e., the proportion of the global sexual minority population who conceals its sexual orientation) using a large sample of sexual minorities collected across 28 countries and an objective index of structural stigma (i.e., discriminatory national laws and policies affecting sexual minorities) across 197 countries. We estimate that the majority (83.0%) of sexual minorities around the world conceal their sexual orientation from all or most people and that country-level structural stigma can serve as a useful predictor of the size of each country’s closeted sexual minority population. Our analysis also predicts that eliminating structural stigma would drastically reduce the size of the global closet. Given its costs to individual health and social equality, the closet represents a considerable burden on the global sexual minority population. The present projection suggests that the surest route to improving the wellbeing of sexual minorities worldwide is through reducing structural forms of inequality. Yet, another route to alleviating the personal and societal toll of the closet is to develop public health interventions that sensitively reach the closeted sexual minority population in high-stigma contexts worldwide. An important goal of this projection, which relies on data from Europe, is to spur future research from non-Western countries capable of refining the estimate of the association between structural stigma and sexual orientation concealment using local experiences of both.

## Introduction

Sexual minorities, in particular thos who identify as lesbian, gay, and bisexual (LGB), represent an increasingly visible segment of the global population. Although the meaning and experience of LGB identification is by no means universal now or historically, global human rights discourse increasingly relies on a shared understanding of LGB identification to achieve its goal of advancing the equal rights and protections of sexual minority populations worldwide.[1] Despite the ability of this globalizing discourse to erase indigenous sexual minority cultures,[2] strong evidence from academic observation, local civil society, and international sexual minority rights organizations suggests that this hegemony has taken hold in all world regions.[2–4] Reflecting this fact, intergovernmental bodies, including the United Nations, have as a goal to “stand up for equal rights and fair treatment for lesbian, gay, bisexual, trans, and intersex people everywhere,”[5] under the assumption that these minority identities and their potential to be stigmatized in fact exist everywhere.

At the same time that LGB identities are increasingly recognized worldwide, the structural treatment of this population varies substantially across countries. On the one hand, among the world’s almost 200 countries, same-sex marriage is now legal in 25, while 43 provide protections against hate crimes committed against sexual minorities.[1] On the other hand, same-sex sexual activity is criminalized in 72 countries and punishable by death in eight of those.[1] In these countries, sexual minority visibility is perilous and open self-expression is often unwise. The extreme variation in country-level laws and policies surrounding sexual minorities can be quantified to produce an index of structural stigma, defined as societal conditions that constrain the opportunities, resources, and well-being of the stigmatized.[6] Recent research shows that the degree of structural stigma surrounding sexual minorities can predict the proportion of sexual minorities who openly express their sexual orientation.[7]

Because country-level variations in structural stigma can predict country-level variations in sexual orientation concealment, and because concealment can exact deep mental and physical health costs and dampen the public visibility necessary for societal change,[8, 9] knowing the size of the global closet represents a public health question with important policy implications. However, a historic lack of cross-national datasets of sexual minorities has precluded accurate estimates of the size of the global closet, here defined as the proportion of the global sexual minority population who conceals its sexual orientation from all or most other people (e.g., family, friends, coworkers, neighbors, medical providers).

To overcome previous barriers to estimating the size of the global closet, we take advantage of the largest known dataset of sexual minority men and women in the world (i.e., the European Union Lesbian, Gay, Bisexual, and Transgender [EU-LGBT] survey), which assess sexual orientation concealment among over 85,000 sexual minorities living across the 28 countries of the European Union.[10] We also capitalize on the ability to extrapolate sexual minorities’ concealment beyond these 28 countries using an objective index of country-level structural stigma in all world countries.[7] Combining these two data sources with indices of other theoretically plausible country-level predictors of sexual orientation concealment (i.e., quality of life, gender inequality, religiosity), we attempted to estimate the proportion of sexual minorities worldwide who currently conceal their sexual orientation from all or most people in their lives. We then estimated the theoretical reduction in the proportion of sexual minorities who would conceal their sexual orientation under future conditions of reduced structural stigma.

While resting on several assumptions regarding the universality of sexual identity experiences, this projection can highlight the approximate proportion of the global sexual minority population that lives in secrecy while motivating targeted intervention approaches to reduce the increasing geographically-contingent inequity in the right to self-expression for sexual minorities worldwide. Given the reliance on extrapolation, results will be interpreted under various possible conditions, including under conditions of no extrapolation (i.e., limited to the observed European data) and in light of limitations in outcome assessment, respondent sampling, and the range of structural stigma represented in the European dataset. This projection responds to globalizing trends in understanding LGB identities, experiences, and rights; estimates one implication of that trend if it continues universally, namely a global closet; and is intended to spur future research from non-Western countries capable of refining the estimate of the association between structural stigma and sexual orientation concealment using local experiences of both.

## Method

### Data sources

The EU-LGBT Survey provided the basis of the study outcome, namely the proportion of sexual minority respondents across the 28 countries of the European Union who reported concealing their sexual orientations from all or most other people in their lives.[10] Several country-level indices were used to predict this outcome, which was then extrapolated across all world countries. The country-level indices included: (1) an index of structural stigma created from an aggregate of both the presence of discriminatory, and absence of protective, national laws and policies,[11] (2) quality of life,[12] (3) gender equality,[13] and (4) religiosity.[14] The study was approved by the Regional Ethics Committee in Stockholm (No. 2017/1852-31/5)

### Outcome variables

#### Sexual orientation concealment

The EU-LGBT Survey was administered between April and July 2012 by the European Union Agency for Fundamental Rights using a web-based survey to assess violations against the fundamental rights of LGBT individuals and their consequences. The survey was developed by a multi-national European team of LGBT topic experts and translated into 27 languages using a comprehensive standardized translation procedure. The final questionnaire was tested for comprehension using cognitive interviews in five countries. Participants from all 28 European Union member countries were recruited online via invitations posted on more than 400 local, national, and international LGBT websites and through social media announcements. Those who fulfilled the eligibility criteria (i.e., residing in one of the 28 EU countries, being at least 18 years of age, and self-identifying as lesbian, gay, bisexual, or transgender) and provided consent to participate were asked to complete the questionnaire on a secure web server. The survey development and methods have been described in detail elsewhere.[10] The analyses presented in the current study were based on information from the 85,582 participants in the survey who identified as lesbian, gay, or bisexual and who completed all study variables. The per-country sample size ranged from 279 participants in Cyprus to 18,760 in Germany. Current analyses utilized the items assessing sexual orientation concealment, which participants completed after reading the following the question: “To how many people among the following groups are you open about yourself being lesbian/gay/bisexual?” using the response options: “none [coded = 3],” “a few [coded = 2],” “most [coded = 1],” “all [coded = 0],” “does not apply to me,” and “don’t know.” Degree of concealment to the following groups of people was assessed: “family members,” “friends,” “neighbors,” “work colleagues/schoolmates,” and “medical staff/health care providers.” Internal consistency across these five items was high (i.e., Cronbach’s α = 0.88). From these items, we calculated two variables: (1) the *proportion of the sexual minority population that reported concealing their sexual orientation*, with those who indicated being open to “none” or “a few” on all items being categorized as “closeted” and those who indicated being open to “most” or “all” on any item categorized as being “open;” (2) the *average degree of sexual orientation concealment among sexual minorities in each country*, based on the average degree of concealment indicated across the five groups of people to whom one could be open.

### Predictor variables

#### Country-level structural stigma

The structural stigma index was created by summing across each of 197 countries’ distinct forms of legal and policy discrimination and protections spanning six domains (i.e., unequal age of consent for same-sex sexual activity, asylum provisions for sexual minorities, protections against bias-motivated violence, legal protections against discrimination, same-sex partnership and parenting recognitions, freedom of assembly) assessed by the International Lesbian, Gay, Bisexual, Trans and Intersex Association.[11] Across these domains, the structural stigma index was created as the sum of up to 15 points for distinct types of discriminatory legislation or loss of up to 12 points for distinct supportive legal protections (see S1 Table for the exact coding). The actual range of this index across the 197 countries in 2017 was 8 to -12, with higher scores indicating higher levels of structural stigma. Since the EU LGBT survey was conducted in 2012, the structural stigma score across the 28 EU member states in 2012 was used in the original prediction model while the 2017 stigma score was used in the extrapolation model across all countries globally. The structural stigma score across EU countries in 2012 was highly correlated with the structural stigma score in 2017 (*r* = 0.88). Across the EU countries, the structural stigma score had improved from 2012 to 2017, with an average reduction of 1.1 (*SD* = 1.4) points.

#### Quality of life

The Human Development Index is a summary measure of quality of life in each world country.[12] It is calculated by the United Nations Development Programme (UNDP) as a composite of indicators of population health and longevity (i.e., life expectancy at birth), education (i.e., expected years of schooling, mean years of schooling), and standard of living (i.e., gross national income per capita). A low value characterizes a country with low quality of life. We used the most recently available data for all countries (i.e., 2015).

#### Gender inequality

The Gender Inequality Index, developed by the UNDP,[13] consists of a composite of indicators of reproductive health (i.e., maternal mortality, adolescent birth rate), power distribution by gender (i.e., female and male population aged 25 years and older with at least secondary education, female and male shares of parliamentary seats), and labor market distribution by gender (i.e., female and male labor force participation rates in the population over age 15).[12, 13] We used the most recently available data for all countries (i.e., 2015).

#### Religiosity

In 2012, Worldwide Independent Network–Gallup International Association, a global network of independent survey organizations, published the *Global Index of Religiosity and Atheism* based on survey results regarding religiosity and atheism across the world.[14] A total of 51,927 individuals from 57 countries across all major world regions completed the survey. Represented countries include: Afghanistan, Argentina, Armenia, Australia, Austria, Azerbaijan, Belgium, Bosnia and Herzegovina, Brazil, Bulgaria, Cameroon, Canada, China, Colombia, Czech Republic, Ecuador, Fiji, Finland, France, Georgia, Germany, Ghana, Hong Kong, Iceland, India, Iraq, Ireland, Italy, Japan, Kenya, South Korea, Lebanon, Lithuania, Macedonia, Malaysia, Moldova, Netherlands, Nigeria, Pakistan, Palestinian territories (West Bank and Gaza), Peru, Poland, Romania, Russian Federation, Saudi Arabia, Serbia, South Africa, South Sudan, Spain, Sweden, Switzerland, Tunisia, Turkey, Ukraine, United States, Uzbekistan, and Vietnam. Between November 2011 and January 2012, a probability sample of approximately 1,000 men and women in each country was interviewed using either face-to-face interview, telephone interview, or web survey. The question used for the current study was: “Irrespective of whether you attend a place of worship or not, would you say you are a religious person, not a religious person, or a convinced atheist?” with the response alternatives: “a religious person,” “not a religious person,” “a convinced atheist,” “don’t know/no response.” Each country was then assigned the value of the proportion of the respondents saying they were a religious person. For those countries not included in the survey, the regional means were imputed depending on their location in one of nine world regions.

#### Country population size

Country population size in 2017 was collected from the World Bank based on counts or estimates of all residents over age 19 regardless of legal status or citizenship.[15] The proportion of LGB-identified sexual minority individuals was assumed to be 3.22% for adult women and 3.37% for adult men based on best-available international estimates.[16]

### Data analysis plan

#### Model 1: Proportion of sexual minority individuals who conceal their sexual orientation extrapolated from country-level structural stigma

To extrapolate the proportion of sexual minority individuals who conceal their sexual orientation in each world country, we first created a regression model using the following function:

$$y_{i}=\beta_{0}+\beta_{1}(structural\;stigma\;2012_{i})+\varepsilon_{i}$$

The observed proportion of sexual minority individuals who concealed their sexual orientation in country *i* in 2012 was regressed onto the structural stigma score for country *i* in 2012. When applied to the EU-LGBT Survey data from 2012, this model explained 53% of the variance in the proportion of sexual minority respondents who conceal their sexual orientation. Next, an estimate of the proportion of sexual minority individuals who concealed their sexual orientation in all world countries was extrapolated from 2017 structural stigma values and the beta estimate derived from Model 1:

$$y_{i}=0.807+0.054(structural\;stigma\;2017_{i})+\varepsilon_{i}$$

#### Model 2: Degree of sexual orientation concealment extrapolated from country-level structural stigma, quality of life, gender equality, and religiosity

To extrapolate sexual minority individuals’ degree of sexual orientation concealment in each world country, we first employed a regression model using the following function:

$$y_{i}=\beta_{0}+\beta_{1}(structural\;stigma\;2012_{i})+\beta_{2}(quality\;of\;life_{i})+\beta_{3}(gender\;inequality_{i})+\beta_{4}(religiosity_{i})+\varepsilon_{i}$$

Degree of sexual orientation concealment in each country *i* in 2012 was regressed onto country-level structural stigma, quality of life, gender inequality, and religiosity. This model explained 89% of the variance in degree of sexual orientation concealment among sexual minorities across European Union countries. Next, the 2017 estimate of degree of concealment among sexual minorities living in all world countries was extrapolated from 2017 structural stigma and the beta estimate identified in the model from the regression analysis:

$$y_{i}=10.502-0.053(structural\;stigma\;2017_{i})-9.819(quality\;of\;life_{i})-1.917(gender\;inequality_{i})+0.006(religiosity_{i})+\varepsilon_{i}$$

The projected values of degree of concealment were not capped at the maximum scale value (i.e., 3) because the range of global structural stigma extended beyond the maximum structural stigma of the 28 EU countries and some countries’ projected degree of concealment thereby could theoretically (and actually) exceed the maximum concealment scale value.

We then attempted to estimate the error associated with our prediction by creating a model based on those EU countries with the lowest structural stigma (*n* = 12), which we then used to predict the values of the remaining countries (*n* = 16). Calculating the percentage prediction error using the equation, [measured value–predicted value] / measured value, yielded a predicted error of 8.6%, suggesting a relatively low prediction error, at least using the observed data.

#### Model 3: Reduction in proportion of closeted sexual minority population if structural stigma were eliminated

Using Model 1, we estimated the reduction in the proportion of sexual minority individuals that would be closeted if structural stigma were eliminated. In these calculations, we assumed that every country would have a score of -12 points on the structural stigma scale.

$$y_{i}=0.807+0.054(-12)=0.159=15.9\%$$

The projected proportion of sexual minorities who conceal their sexual orientation was constrained to fall within 0 to 100% of the population.

## Results

Model 1 estimates that the global proportion of the sexual minority population that conceals its sexual orientation from all or most people is 83.0%. The model estimates that sexual minorities in the Middle East and North African (94.8%) and Sub-Saharan African (89.5%) regions are most likely to be closeted whereas those in the Latin American and Caribbean (35.4%) and Northern/Western European (36.5%**)** region are least likely to be closeted.

**Fig 1:**
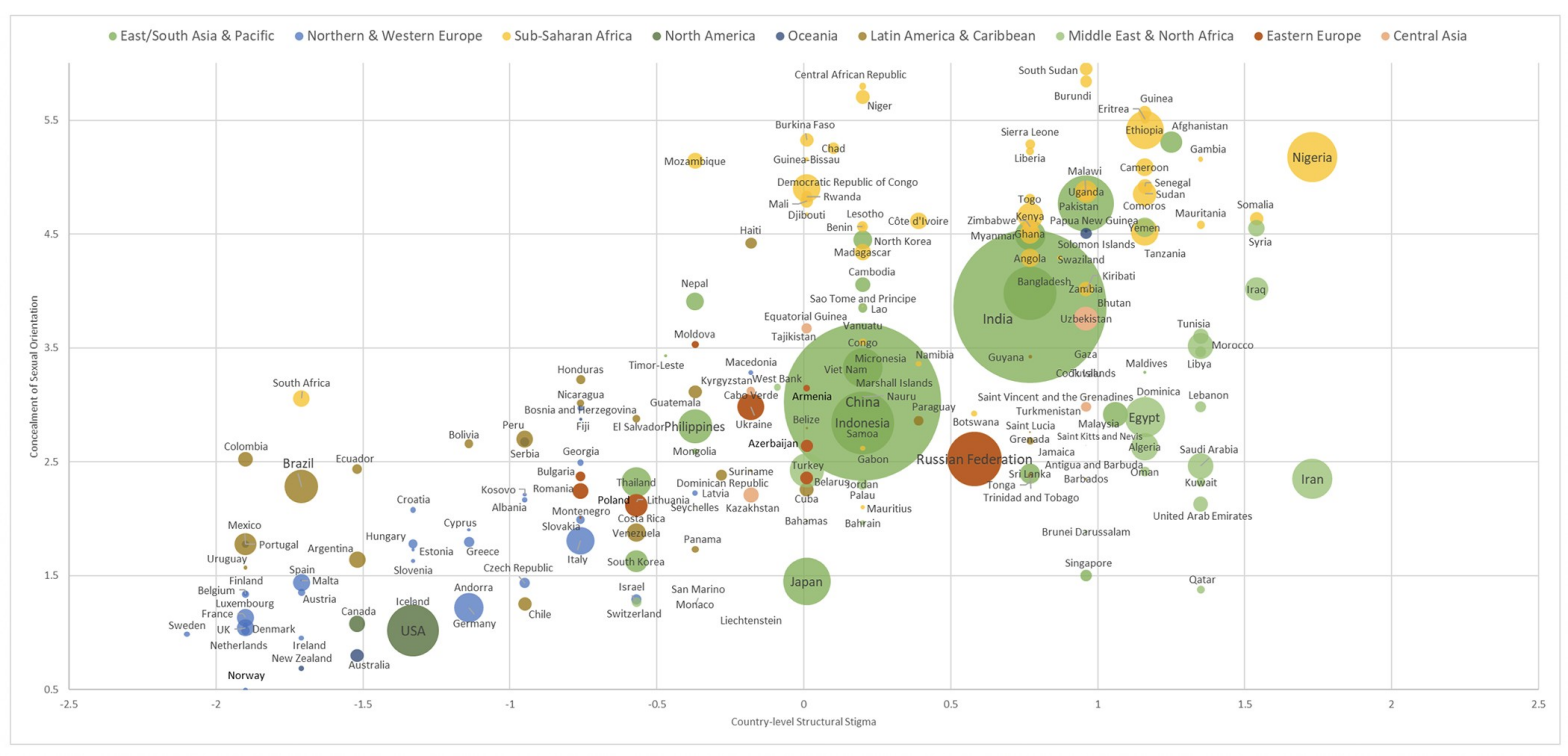
Degree of concealment of sexual orientation among lesbian, gay, and bisexual individuals globally by country-level structural stigma (size of circles is relative to size of closeted sexual minority population in each country). The projected values of degree of concealment were not capped at the maximum scale value (i.e., 3) because the range of global structural stigma extended beyond the maximum structural stigma of the 28 EU countries and some countries’ projected degree of concealment thereby could theoretically (and actually) exceed the maximum concealment scale value.

Fig 1 shows our estimate of both the size of the sexual minority population that conceals its sexual orientation (Model 1) and the degree of concealment among sexual minorities (Model 2) as a function of country-level structural stigma in 197 counties globally in 2017. In the figure, each country’s level of structural stigma is reported on the *x*-axis and each country’s average degree of sexual orientation concealment on the *y*-axis. The size of the closeted sexual minority population is illustrated by the size of each country’s circle. Degree of concealment was highest in certain African and Middle Eastern countries and lowest in Western Europe, North America, Australia, and New Zealand. S2 Table shows the structural stigma and concealment scores for each country.

**Fig 2:**
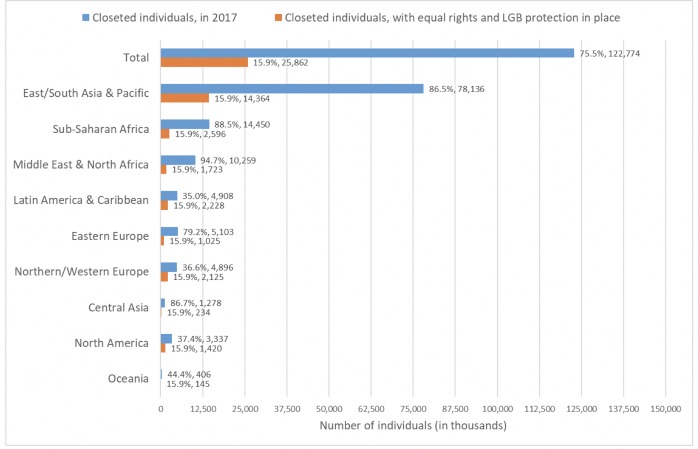
Expected reduction in proportion and number of closeted sexual minority individuals if structural stigma were eliminated. Simulation results, aggregated by region, of number of closeted sexual minority individuals globally in 2017 (blue bars) and if structural stigma were eliminated (orange bars).

Fig 2 illustrates, according to world region, the reduction in the proportion of the sexual minority population that would conceal its sexual orientation if structural stigma were eliminated. While the top bar shows that 83.0% of the global sexual minority population is estimated to have concealed its sexual orientation from all or most other individuals in 2017, the bottom bar shows that 15.9% would be expected to do so with the complete elimination of structural stigma.

## Discussion

Responding to the increasingly prominent assumption that sexual minority identities are similarly legible worldwide and the increasing reliance on this assumption to advance the equal treatment of this population,[1, 5] we set out to estimate one logical corollary of this trend, namely that a proportion of the global sexual minority population will conceal their sexual orientation. Extrapolating from the largest known sample of sexual minority men and women, collected across 28 countries, and an objective index of the country-level structural context surrounding sexual minorities in all world countries, we estimate that the majority of sexual minorities around the world conceal their sexual orientation from all or most family, friends, colleagues, neighbors, and medical providers. Our projection suggests that country-level structural stigma, namely discriminatory laws and policies denying sexual minorities equal rights, can serve as a useful predictor of the size of each country’s closeted sexual minority population. Our projection also suggests that eliminating structural stigma would drastically reduce the size of the global closet.

The reality facing sexual minorities today of being able to be closeted or open, or anywhere in between, is a relatively recent phenomenon, having only emerged around the 20^th^ century with the emergence of public sexual identities in Western countries.[17] Further, contemporary Western research highlights increasing lifespan fluidity of sexual identities among younger cohorts,[18, 19] suggesting that disclosure of a sexual minority identity might not forever remain a singular or even legible event. Nonetheless, the present research suggests that for sexual minorities around the world today, the structural context under which they live can be plausibly used to predict whether they are in or out of the closet. For example, in India, strong pressures, including threats of imprisonment, harassment, blackmail, and sexual assault, face sexual minorities who are open about their sexual orientation.[20] In China, sexual minorities have no legal recourse against targeted discrimination and harassment; harmful sexual orientation conversion therapies are normative.[21] The present research suggests that sexual orientation concealment is also normative in these, and most other, countries as a function of such structural realities.

While sexual orientation concealment can serve as a functional adaptation to hostile structural contexts, it can nevertheless exact negative mental and physical health–and societal–costs. Sexual orientation concealment has been linked to depressive and anxious symptoms, substance abuse, relationship difficulties, infectious disease susceptibility, and premature mortality.[8, 22] Insidiously, sexual orientation concealment can also perpetuate structural inequality. Open self-expression of one’s sexual orientation can elicit familiarity, acceptance, and ultimately the public support required for structural reform.[9, 23] Conversely, as a recursive mechanism through which structural stigma operates, sexual orientation concealment keeps sexual minorities underground and invisible to mainstream society, which in turn hampers structural change. By highlighting the considerable size of the global closet, the present study underscores the equally considerable cost of sexual orientation concealment to individual health and social equality around the world. While reductions in structural stigma have been shown to partially alleviate the health burden facing sexual minorities,[24, 25] whether this effect operates through parallel reductions in sexual orientation concealment remains to be determined. Cross-sectional research demonstrating that sexual orientation concealment mediates the association between structural stigma and life satisfaction and, among sexual minority men, HIV-risk prevention,[7, 26] provides preliminary support for this possibility.

Given that the broad goals of this study necessarily rely on a large degree of extrapolation from the observed data, study results should be parameterized. Limiting results to the observed data, here collected from the 28 European Union countries, demands the smallest degree of inference and therefore yields the most reliable estimate of closet size. Even from the observed data alone, we find that four-fifths of sexual minorities living in Eastern Europe report concealing their sexual orientation from all or most other individuals. This situation differs substantially from that in Northern/Western Europe in which only about one-third reports the same.

Extrapolating the global closet size requires several significant assumptions that could have led our models to either over- or under-estimate the size of the closet if shown to be false. While sexual minorities exist across the world, the experience of minority sexual identities is shaped by local norms, practices, and ideologies.[27] While our projection responds to the increasing assumption of similarity of sexual minority identities and experiences across countries,[1–5] we of course recognize that some cultures draw upon an indigenous understanding of sexuality that does not overlap with hegemonic Western understandings of “lesbian,” “bisexual,” or “gay.” For instance, hijra individuals of South Asia are recognized as being neither male nor female but occupying characteristics of both genders; they might renounce a sexual identity altogether.[28] Further, in many societies, the insertive male partner in sexual intercourse with other men does not occupy a distinct sexual minority identity. Because such identities are not captured in the EU-LGBT Survey that serves as the starting point of our extrapolation, our estimates cannot apply to these populations whose experience of labeling, and concealing or disclosing, these identities might differ from those with identities of “lesbian,” “gay,” and “bisexual.” It is also possible that our projection is confounded by a potential tendency for sexual minorities in high-stigma countries to experience relatively lower access to “lesbian,” “gay,” or “bisexual” identities, thereby producing another source of over-estimation of the global closet size as estimated from these identities. However, given substantial evidence suggesting that sexual minorities exist in all regions of the world[27] and that cultural differences in the experience of sexual minority status might simply represent variations around a common theme,[4] our estimates should at least roughly apply. We also intend for this initial projection to spur future research from non-Western countries capable of refining the estimate of the association between structural stigma and sexual orientation concealment using local experiences of both.

Another potential source of overestimation stems from our outcome assessment. Specifically, our assessment of the closet derives from a one-item question regarding the degree to which one is open about their sexual orientation to five groups of people (i.e., family, friends, colleagues, neighbors, and medical providers). Because these categories are not exhaustive, sexual minorities who are open to other individuals not assessed here (e.g., romantic or sexual partners) would nonetheless be categorized as closeted in our analyses. Future research is needed to examine the validity of this item for capturing individuals who are closeted. Also, because we considered as closeted those sexual minorities who are open to “a few” people in their lives about their sexual orientation, it is possible that some individuals considered closeted in our estimate are actually open to a few essential people in their lives.

Several other assumptions might have led our model to underestimate the global closet size. First, the EU-LGBT Survey likely disproportionately samples open sexual minorities, given the focus of the survey. In fact, a recent community survey in Canada finds that 30% of gay and bisexual men would not disclosure their sexual orientation on a government-administered survey.[29] While similar estimates of unwillingness to disclose on surveys do not exist for sexual minority women, this finding suggests a potential need to upwardly revise our estimate of the global closet size. Second, some individuals engage in same-sex sexual behavior but do not identify as a sexual minority, therefore suggesting the possible need to revise our estimate upward still. However, data from a very large survey of men who have sex with men across 38 countries finds that only a small proportion (less than 1.0%) of this sample does not identify as gay or bisexual.[30] Also, the proportion of closeted sexual minorities found in low-stigma countries in the EU-LGBT Survey roughly parallels the proportion of this population found in the only two known population-based surveys of the closet, also conducted in low-stigma contexts (i.e., California and Sweden).[31, 32] Another source of estimation imprecision stems from range restriction in our initial predictor. While highly variable, the range of structural stigma across European countries does not cover the upper limit of structural stigma globally; thus, the validity of our extrapolation outside of the range of European structural stigma is uncertain. However, we hypothesize that the association between country-level structural stigma and concealment is curvilinear such that at the highest levels of stigma, the linear trend would plateau. That is, we hypothesize, for example, that in countries that issue a legal sentence for homosexuality, whether that sentence is two years (receiving 1 points according to our structural stigma measure) or death (receiving 5 points according to our structural stigma measure) is not likely to appreciably change one’s likelihood of concealment. Finally, the EU-LGBT Survey sample was limited to adults above age 18, thereby not capturing sexual minority youth and adolescents who, by virtue of the developmental timing of the closet and coming out, are more likely to be closeted than adults.[33] The above evidence, when taken in aggregate, suggests that the estimate of the size of the global closet found here might underestimate its true size.

Assuming continued globalizing understandings of sexual minority identities worldwide[1, 5] and permitting even wide error in this first extrapolation of the size of the global closet, the results of the present study suggest that a majority of sexual minorities worldwide are not open about their sexual orientation to most or all people in their lives. Given its costs to individual health and social equality, the closet, according to our projection, represents a considerable burden on the global sexual minority population. The present estimates suggest that the surest route to improving the wellbeing of sexual minorities worldwide is through reducing structural forms of inequality through national legislation. Until full equality is achieved, another route to alleviating the personal and societal toll of the closet is to develop public health interventions that sensitively reach, and address the needs of, the closeted sexual minority population and to deliver such interventions using efficient means. Because high-stigma locales are particularly likely to both perpetuate the closet and to lack the affirmative resources necessary for reaching the closeted population, technology-assisted interventions might be especially warranted to empower both sexual minorities and the public health practitioners who could serve as allies in efforts to improve the wellbeing, equality, and dignity of this sizable segment of the global population.[34, 35].

## Supporting information

S1 Table Coding of structural stigma based on discriminatory legislation, recognition of same-sex relationships, and protection against discrimination, for the 197 countries included in the study. (DOCX)

S2 Table Data used to illustrate country-level associations between structural stigma and sexual orientation concealment globally (Fig 1), including global region; International Lesbian, Gay, Bisexual, Trans, and Intersex Association (ILGA) score; degree of sexual orientation concealment; and total number of sexual minorities concealing their sexual orientation across 197 countries. The projected values of degree of concealment were not capped at the maximum scale value (i.e., 3) because the range of global structural stigma extended beyond the maximum structural stigma of the 28 EU countries and some countries’ projected degree of concealment thereby could theoretically (and actually) exceed the maximum concealment scale value. The projected proportion of sexual minorities who conceal their sexual orientation was constrained to fall within 0 to 100% of the population.
(DOCX)
