# Supplementary material for: How many sexual minorities are hidden? Projecting the size of the global closet with implications for policy and public health
Source: PLoS One. 2019 Jun 13;14(6):e0218084. doi: 10.1371/journal.pone.0218084 (PMC6564426; doi:10.1371/journal.pone.0218084)
Supplement: S1 Table — (DOCX) [file pone.0218084.s001.docx]

**S1 Table**

| **Legal discrimination and criminalization** | |
| --- | --- |
|  | Equal age of consent for same-sex and opposite-sex sexual acts (-1 point)  Illegality of male homosexuality (1 point)  Illegality of female homosexuality (1 point)  Any arrest due to homosexuality in past 3 years (1 point)  Presence of penalizing legal texts:   - Sexual acts (1 point) - Sodomy (1 point) - Against nature (1 point) - Buggery (1 point) - Indecency/other (1 point)   Presence of morality codes:   - Penal code (1 point) - Morality code (1 point)   Criminal sentences issued:   - 1 month to 2 years (1 point) *or* - 3 to 7 years (2 points) *or* - 8 to 13 years (3 points) *or* - 14 – years to life (4 points) *or* - Death (5 points) |
| **Recognition** | |
|  | Equal rights included in constitution (-1 point)  Same-sex civil relationships legally recognized (-2 points)  Same-sex marriages legally recognized (-3 points)  Joint adoption legally permitted for same-sex couples (-1 point)  Second parent adoption legally permitted for same-sex couples (-1 point) |
| **Protection** | |
|  | Presence of employment protections (-1 point)  Presence of hate crimes protections present (-1 point)  Presence of incitement protections present (-1 point) |
| **Total points** | |
|  | Maximum high country-level structural stigma score: 15  Maximum low country-level structural stigma score: -12 |
