# Supplementary material for: How many sexual minorities are hidden? Projecting the size of the global closet with implications for policy and public health
Source: PLoS One. 2019 Jun 13;14(6):e0218084. doi: 10.1371/journal.pone.0218084 (PMC6564426; doi:10.1371/journal.pone.0218084)
Supplement: S2 Table — The projected values of degree of concealment were not capped at the maximum scale value (i.e., 3) because the range of global structural stigma extended beyond the maximum structural stigma of the 28 EU countries and some countries’ projected degree of concealment thereby could theoretically (and actually) exceed the maximum concealment scale value. The projected proportion of sexual minorities who conceal their sexual orientation was constrained to fall within 0 to 100% of the population. (DOCX) [file pone.0218084.s002.docx]

**S2 Table**

| Country | Region | Structural stigma  (z-score) | Concealment of  sexual orientation  (95% confidence intervals) | | Proportion of sexual minorities concealing their sexual orientation (95% confidence intervals) | | Number of sexual minorities concealing their sexual orientation |
| --- | --- | --- | --- | --- | --- | --- | --- |
| Afghanistan | East/South Asia & Pacific | 1.25 | 5.31 | (4.97; 5.65) | 100% | (72%; 100%) | 526097 |
| Albania | Northern & Western Europe | -0.95 | 2.17 | (1.83; 2.51) | 48% | (20%; 76%) | 33940 |
| Algeria | Middle East & North Africa | 1.16 | 2.63 | (2.29; 2.97) | 100% | (72%; 100%) | 868259 |
| Andorra | Northern & Western Europe | -1.14 | 1.37 | (1.03; 1.71) | 43% | (15%; 71%) | 640 |
| Angola | Sub-Saharan Africa | 0.77 | 4.29 | (3.95; 4.63) | 97% | (69%; 100%) | 405752 |
| Antigua and Barbuda | Latin America & Caribbean | 0.96 | 2.44 | (2.10; 2.78) | 100% | (72%; 100%) | 2272 |
| Argentina | Latin America & Caribbean | -1.52 | 1.64 | (1.30; 1.98) | 32% | (4%; 60%) | 313620 |
| Armenia | Eastern Europe | 0.01 | 3.15 | (2.81; 3.49) | 75% | (47%; 100%) | 53588 |
| Australia | Oceania | -1.52 | 0.80 | (0.46; 1.14) | 32% | (4%; 60%) | 194326 |
| Austria | Northern & Western Europe | -1.71 | 1.36 | (1.02; 1.70) | 27% | (0%; 55%) | 63261 |
| Azerbaijan | Eastern Europe | 0.01 | 2.64 | (2.30; 2.98) | 75% | (47%; 100%) | 170116 |
| Bahamas | Latin America & Caribbean | 0.01 | 1.98 | (1.64; 2.32) | 75% | (47%; 100%) | 7067 |
| Bahrain | Middle East & North Africa | 0.2 | 1.96 | (1.62; 2.30) | 81% | (53%; 100%) | 30101 |
| Bangladesh | East/South Asia & Pacific | 0.77 | 3.98 | (3.64; 4.32) | 97% | (69%; 100%) | 3252286 |
| Barbados | Latin America & Caribbean | 0.96 | 2.35 | (2.01; 2.69) | 100% | (72%; 100%) | 7004 |
| Belarus | Eastern Europe | 0.01 | 2.36 | (2.02; 2.70) | 75% | (47%; 100%) | 184161 |
| Belgium | Northern & Western Europe | -1.9 | 1.34 | (1.00; 1.68) | 21% | (0%; 49%) | 60923 |
| Belize | Latin America & Caribbean | 0.01 | 2.80 | (2.46; 3.14) | 75% | (47%; 100%) | 5375 |
| Benin | Sub-Saharan Africa | 0.2 | 4.56 | (4.22; 4.90) | 81% | (53%; 100%) | 139081 |
| Bhutan | East/South Asia & Pacific | 0.96 | 3.84 | (3.50; 4.18) | 100% | (72%; 100%) | 17136 |
| Bolivia | Latin America & Caribbean | -1.14 | 2.66 | (2.32; 3.00) | 43% | (15%; 71%) | 91443 |
| Bosnia and Herzegovina | Northern & Western Europe | -0.76 | 2.97 | (2.63; 3.31) | 54% | (26%; 82%) | 49787 |
| Botswana | Sub-Saharan Africa | 0.58 | 2.92 | (2.58; 3.26) | 92% | (64%; 100%) | 41133 |
| Brazil | Latin America & Caribbean | -1.71 | 2.28 | (1.94; 2.62) | 27% | (0%; 55%) | 1304072 |
| Brunei Darussalam | East/South Asia & Pacific | 0.96 | 1.89 | (1.55; 2.23) | 100% | (72%; 100%) | 9714 |
| Bulgaria | Eastern Europe | -0.76 | 2.37 | (2.03; 2.71) | 54% | (26%; 82%) | 102611 |
| Burkina Faso | Sub-Saharan Africa | 0.01 | 5.33 | (4.99; 5.67) | 75% | (47%; 100%) | 208151 |
| Burundi | Sub-Saharan Africa | 0.96 | 5.84 | (5.50; 6.18) | 100% | (72%; 100%) | 161139 |
| Cabo Verde | Sub-Saharan Africa | -0.18 | 3.09 | (2.75; 3.43) | 70% | (42%; 98%) | 7487 |
| Cambodia | East/South Asia & Pacific | 0.2 | 4.05 | (3.71; 4.39) | 81% | (53%; 100%) | 253149 |
| Cameroon | Sub-Saharan Africa | 1.16 | 5.09 | (4.75; 5.43) | 100% | (72%; 100%) | 370510 |
| Canada | North America | -1.52 | 1.08 | (0.74; 1.42) | 32% | (4%; 60%) | 303382 |
| Central African Republic | Sub-Saharan Africa | 0.2 | 5.80 | (5.46; 6.14) | 81% | (53%; 100%) | 56245 |
| Chad | Sub-Saharan Africa | 0.1 | 5.25 | (4.91; 5.59) | 78% | (50%; 100%) | 159453 |
| Chile | Latin America & Caribbean | -0.95 | 1.25 | (0.91; 1.59) | 48% | (20%; 76%) | 206833 |
| China | East/South Asia & Pacific | 0.2 | 3.03 | (2.69; 3.37) | 81% | (53%; 100%) | 28422500 |
| Colombia | Latin America & Caribbean | -1.9 | 2.52 | (2.18; 2.86) | 21% | (0%; 49%) | 231615 |
| Comoros | Sub-Saharan Africa | 1.16 | 4.75 | (4.41; 5.09) | 100% | (72%; 100%) | 13372 |
| Congo | Sub-Saharan Africa | 0.2 | 3.55 | (3.21; 3.89) | 81% | (53%; 100%) | 67009 |
| Democratic Republic of Congo | Sub-Saharan Africa | 0.01 | 4.90 | (4.56; 5.24) | 75% | (47%; 100%) | 871274 |
| Cook Islands | Oceania | 0.96 | 3.28 | (2.94; 3.62) | 100% | (72%; 100%) | 335 |
| Costa Rica | Latin America & Caribbean | -0.57 | 2.08 | (1.74; 2.42) | 59% | (31%; 87%) | 67262 |
| Côte d'Ivoire | Sub-Saharan Africa | 0.39 | 4.62 | (4.28; 4.96) | 86% | (58%; 100%) | 321280 |
| Croatia | Northern & Western Europe | -1.33 | 2.08 | (1.74; 2.42) | 38% | (10%; 66%) | 41226 |
| Cuba | Latin America & Caribbean | 0.01 | 2.26 | (1.92; 2.60) | 75% | (47%; 100%) | 221726 |
| Cyprus | Northern & Western Europe | -1.14 | 1.90 | (1.56; 2.24) | 43% | (15%; 71%) | 12813 |
| Czech Republic | Northern & Western Europe | -0.95 | 1.44 | (1.10; 1.78) | 48% | (20%; 76%) | 134656 |
| Denmark | Northern & Western Europe | -1.9 | 1.07 | (0.73; 1.41) | 21% | (0%; 49%) | 30865 |
| Djibouti | Sub-Saharan Africa | 0.01 | 4.67 | (4.33; 5.01) | 75% | (47%; 100%) | 13876 |
| Dominica | Latin America & Caribbean | 1.16 | 3.08 | (2.74; 3.42) | 100% | (72%; 100%) | 1416 |
| Dominican Republic | Latin America & Caribbean | -0.28 | 2.38 | (2.04; 2.72) | 67% | (39%; 95%) | 146269 |
| Ecuador | Latin America & Caribbean | -1.52 | 2.44 | (2.10; 2.78) | 32% | (4%; 60%) | 109605 |
| Egypt | Middle East & North Africa | 1.16 | 2.89 | (2.55; 3.23) | 100% | (72%; 100%) | 1864151 |
| El Salvador | Latin America & Caribbean | -0.57 | 2.88 | (2.54; 3.22) | 59% | (31%; 87%) | 77392 |
| Equatorial Guinea | Sub-Saharan Africa | 0.01 | 3.69 | (3.35; 4.03) | 75% | (47%; 100%) | 16924 |
| Eritrea | Sub-Saharan Africa | 1.16 | 5.51 | (5.17; 5.85) | 100% | (72%; 100%) | 86169 |
| Estonia | Northern & Western Europe | -1.33 | 1.73 | (1.39; 2.07) | 38% | (10%; 66%) | 13042 |
| Ethiopia | Sub-Saharan Africa | 1.16 | 5.41 | (5.07; 5.75) | 100% | (72%; 100%) | 1649584 |
| Fiji | Oceania | -0.76 | 2.87 | (2.53; 3.21) | 54% | (26%; 82%) | 10168 |
| Finland | Northern & Western Europe | -1.9 | 1.35 | (1.01; 1.69) | 21% | (0%; 49%) | 29796 |
| France | Northern & Western Europe | -1.9 | 1.13 | (0.79; 1.47) | 21% | (0%; 49%) | 352467 |
| Gabon | Sub-Saharan Africa | 0.2 | 2.62 | (2.28; 2.96) | 81% | (53%; 100%) | 29695 |
| Gambia | Sub-Saharan Africa | 1.35 | 5.16 | (4.82; 5.50) | 100% | (72%; 100%) | 30277 |
| Gaza | Middle East & North Africa | 0.96 | 3.45 | (3.11; 3.79) | 100% | (72%; 100%) | 35623 |
| Georgia | Northern & Western Europe | -0.76 | 2.49 | (2.15; 2.83) | 54% | (26%; 82%) | 49826 |
| Germany | Northern & Western Europe | -1.14 | 1.22 | (0.88; 1.56) | 43% | (15%; 71%) | 960526 |
| Ghana | Sub-Saharan Africa | 0.77 | 4.51 | (4.17; 4.85) | 97% | (69%; 100%) | 471786 |
| Greece | Northern & Western Europe | -1.14 | 1.80 | (1.46; 2.14) | 43% | (15%; 71%) | 123061 |
| Grenada | Latin America & Caribbean | 0.77 | 2.70 | (2.36; 3.04) | 97% | (69%; 100%) | 2246 |
| Guatemala | Latin America & Caribbean | -0.37 | 3.12 | (2.78; 3.46) | 65% | (37%; 93%) | 194703 |
| Guinea | Sub-Saharan Africa | 1.16 | 5.57 | (5.23; 5.91) | 100% | (72%; 100%) | 197165 |
| Guinea-Bissau | Sub-Saharan Africa | 0.01 | 5.16 | (4.82; 5.50) | 75% | (47%; 100%) | 22134 |
| Guyana | Latin America & Caribbean | 0.77 | 3.42 | (3.08; 3.76) | 97% | (69%; 100%) | 15045 |
| Haiti | Latin America & Caribbean | -0.18 | 4.42 | (4.08; 4.76) | 70% | (42%; 98%) | 143739 |
| Honduras | Latin America & Caribbean | -0.76 | 3.22 | (2.88; 3.56) | 54% | (26%; 82%) | 94954 |
| Hungary | Northern & Western Europe | -1.33 | 1.78 | (1.44; 2.12) | 38% | (10%; 66%) | 98659 |
| Iceland | Northern & Western Europe | -1.33 | 1.28 | (0.94; 1.62) | 38% | (10%; 66%) | 3162 |
| India | East/South Asia & Pacific | 0.77 | 3.86 | (3.52; 4.20) | 97% | (69%; 100%) | 26942326 |
| Indonesia | East/South Asia & Pacific | 0.2 | 2.84 | (2.50; 3.18) | 81% | (53%; 100%) | 4506845 |
| Iran | Middle East & North Africa | 1.73 | 2.35 | (2.01; 2.69) | 100% | (72%; 100%) | 1867350 |
| Iraq | Middle East & North Africa | 1.54 | 4.02 | (3.68; 4.36) | 100% | (72%; 100%) | 620999 |
| Ireland | Northern & Western Europe | -1.71 | 0.95 | (0.61; 1.29) | 27% | (0%; 55%) | 30961 |
| Israel | Middle East & North Africa | -0.57 | 1.27 | (0.93; 1.61) | 59% | (31%; 87%) | 109008 |
| Italy | Northern & Western Europe | -0.76 | 1.81 | (1.47; 2.15) | 54% | (26%; 82%) | 880003 |
| Jamaica | Latin America & Caribbean | 0.77 | 2.68 | (2.34; 3.02) | 97% | (69%; 100%) | 63050 |
| Japan | East/South Asia & Pacific | 0.01 | 1.45 | (1.11; 1.79) | 75% | (47%; 100%) | 2582872 |
| Jordan | Middle East & North Africa | 0.2 | 2.31 | (1.97; 2.65) | 81% | (53%; 100%) | 141385 |
| Kazakhstan | Central Asia | -0.18 | 2.21 | (1.87; 2.55) | 70% | (42%; 98%) | 275050 |
| Kenya | Sub-Saharan Africa | 0.77 | 4.66 | (4.32; 5.00) | 97% | (69%; 100%) | 770993 |
| Kiribati | Oceania | 0.96 | 4.00 | (3.66; 4.34) | 100% | (72%; 100%) | 2120 |
| Kosovo | Northern & Western Europe | -0.95 | 2.21 | (1.87; 2.55) | 48% | (20%; 76%) | 16787 |
| Kuwait | Middle East & North Africa | 1.35 | 2.32 | (1.98; 2.66) | 100% | (72%; 100%) | 100067 |
| Kyrgyzstan | Central Asia | -0.18 | 3.12 | (2.78; 3.46) | 70% | (42%; 98%) | 86022 |
| Lao | East/South Asia & Pacific | 0.2 | 3.85 | (3.51; 4.19) | 81% | (53%; 100%) | 103983 |
| Latvia | Northern & Western Europe | -0.37 | 2.22 | (1.88; 2.56) | 65% | (37%; 93%) | 33361 |
| Lebanon | Middle East & North Africa | 1.35 | 2.98 | (2.64; 3.32) | 100% | (72%; 100%) | 136227 |
| Lesotho | Sub-Saharan Africa | 0.2 | 4.56 | (4.22; 4.90) | 81% | (53%; 100%) | 31879 |
| Liberia | Sub-Saharan Africa | 0.77 | 5.23 | (4.89; 5.57) | 97% | (69%; 100%) | 71725 |
| Libya | Middle East & North Africa | 1.35 | 3.46 | (3.12; 3.80) | 100% | (72%; 100%) | 133209 |
| Liechtenstein | Northern & Western Europe | -0.18 | 1.11 | (0.77; 1.45) | 70% | (42%; 98%) | 508 |
| Lithuania | Northern & Western Europe | -0.57 | 2.14 | (1.80; 2.48) | 59% | (31%; 87%) | 43848 |
| Luxembourg | Northern & Western Europe | -1.9 | 1.26 | (0.92; 1.60) | 21% | (0%; 49%) | 3202 |
| Madagascar | Sub-Saharan Africa | 0.2 | 4.34 | (4.00; 4.68) | 81% | (53%; 100%) | 327014 |
| Malawi | Sub-Saharan Africa | 0.96 | 4.86 | (4.52; 5.20) | 100% | (72%; 100%) | 274717 |
| Malaysia | East/South Asia & Pacific | 1.06 | 2.92 | (2.58; 3.26) | 100% | (72%; 100%) | 697178 |
| Maldives | East/South Asia & Pacific | 1.16 | 3.29 | (2.95; 3.63) | 100% | (72%; 100%) | 10089 |
| Mali | Sub-Saharan Africa | 0.01 | 4.79 | (4.45; 5.13) | 75% | (47%; 100%) | 190452 |
| Malta | Northern & Western Europe | -1.71 | 1.46 | (1.12; 1.80) | 27% | (0%; 100%) | 3345 |
| Marshall Islands | Oceania | 0.2 | 3.07 | (2.73; 3.41) | 81% | (53%; 100%) | 828 |
| Mauritania | Sub-Saharan Africa | 1.35 | 4.58 | (4.24; 4.92) | 100% | (72%; 100%) | 72608 |
| Mauritius | Sub-Saharan Africa | 0.2 | 2.10 | (1.76; 2.44) | 81% | (53%; 100%) | 25022 |
| Mexico | Latin America & Caribbean | -1.9 | 1.78 | (1.44; 2.12) | 21% | (0%; 49%) | 573899 |
| Micronesia | Oceania | 0.2 | 3.30 | (2.96; 3.64) | 81% | (53%; 100%) | 1549 |
| Moldova | Eastern Europe | -0.37 | 3.53 | (3.19; 3.87) | 65% | (37%; 93%) | 59886 |
| Monaco | Northern & Western Europe | -0.37 | 1.25 | (0.91; 1.59) | 65% | (37%; 93%) | 482 |
| Mongolia | East/South Asia & Pacific | -0.37 | 2.59 | (2.25; 2.93) | 65% | (37%; 93%) | 41663 |
| Montenegro | Eastern Europe | -0.76 | 2.01 | (1.67; 2.35) | 54% | (26%; 82%) | 8349 |
| Morocco | Middle East & North Africa | 1.35 | 3.52 | (3.18; 3.86) | 100% | (72%; 100%) | 757947 |
| Mozambique | Sub-Saharan Africa | -0.37 | 5.14 | (4.80; 5.48) | 65% | (37%; 93%) | 281024 |
| Myanmar | East/South Asia & Pacific | 0.77 | 4.49 | (4.15; 4.83) | 97% | (69%; 100%) | 1087226 |
| Namibia | Sub-Saharan Africa | 0.39 | 3.36 | (3.02; 3.70) | 86% | (58%; 100%) | 37820 |
| Nauru | Oceania | 0.2 | 3.07 | (2.73; 3.41) | 81% | (53%; 100%) | 203 |
| Nepal | East/South Asia & Pacific | -0.37 | 3.91 | (3.57; 4.25) | 65% | (37%; 93%) | 362138 |
| Netherlands | Northern & Western Europe | -1.9 | 1.02 | (0.68; 1.36) | 21% | (0%; 49%) | 92004 |
| New Zealand | Oceania | -1.71 | 0.69 | (0.35; 1.03) | 27% | (0%; 55%) | 31386 |
| Nicaragua | Latin America & Caribbean | -0.76 | 3.02 | (2.68; 3.36) | 54% | (26%; 82%) | 67867 |
| Niger | Sub-Saharan Africa | 0.2 | 5.71 | (5.37; 6.05) | 81% | (53%; 100%) | 224625 |
| Nigeria | Sub-Saharan Africa | 1.73 | 5.18 | (4.84; 5.52) | 100% | (72%; 100%) | 2868831 |
| North Korea | East/South Asia & Pacific | 0.2 | 4.45 | (4.11; 4.79) | 81% | (53%; 100%) | 395675 |
| Norway | Northern & Western Europe | -1.9 | 0.50 | (0.16; 0.84) | 21% | (0%; 49%) | 27780 |
| Oman | Middle East & North Africa | 1.16 | 2.41 | (2.07; 2.75) | 100% | (72%; 100%) | 112093 |
| Pakistan | East/South Asia & Pacific | 0.96 | 4.77 | (4.43; 5.11) | 100% | (72%; 100%) | 3600873 |
| Palau | Oceania | 0.2 | 2.21 | (1.87; 2.55) | 81% | (53%; 100%) | 335 |
| Panama | Latin America & Caribbean | -0.37 | 1.73 | (1.39; 2.07) | 65% | (37%; 93%) | 56260 |
| Papua New Guinea | Oceania | 0.96 | 4.51 | (4.17; 4.85) | 100% | (72%; 100%) | 145912 |
| Paraguay | Latin America & Caribbean | 0.39 | 2.86 | (2.52; 3.20) | 86% | (58%; 100%) | 117241 |
| Peru | Latin America & Caribbean | -0.95 | 2.70 | (2.36; 3.04) | 48% | (20%; 76%) | 325438 |
| Philippines | East/South Asia & Pacific | -0.37 | 2.81 | (2.47; 3.15) | 65% | (37%; 93%) | 1314375 |
| Poland | Eastern Europe | -0.57 | 2.12 | (1.78; 2.46) | 59% | (31%; 87%) | 591858 |
| Portugal | Northern & Western Europe | -1.9 | 1.78 | (1.44; 2.12) | 21% | (0%; 49%) | 57721 |
| Qatar | Middle East & North Africa | 1.35 | 1.38 | (1.04; 1.72) | 100% | (72%; 100%) | 71934 |
| Romania | Eastern Europe | -0.76 | 2.24 | (1.90; 2.58) | 54% | (26%; 82%) | 276197 |
| Russian Federation | Eastern Europe | 0.58 | 2.53 | (2.19; 2.87) | 92% | (64%; 100%) | 3408895 |
| Rwanda | Sub-Saharan Africa | 0.01 | 4.83 | (4.49; 5.17) | 75% | (47%; 100%) | 148860 |
| Saint Kitts and Nevis | Latin America & Caribbean | 0.96 | 2.64 | (2.30; 2.98) | 100% | (72%; 100%) | 1056 |
| Saint Lucia | Latin America & Caribbean | 0.77 | 2.76 | (2.42; 3.10) | 97% | (69%; 100%) | 4157 |
| Saint Vincent and the Grenadines | Latin America & Caribbean | 0.96 | 3.07 | (2.73; 3.41) | 100% | (72%; 100%) | 2451 |
| Samoa | Oceania | 0.2 | 2.75 | (2.41; 3.09) | 81% | (53%; 100%) | 2775 |
| San Marino | Northern & Western Europe | -0.37 | 1.25 | (0.91; 1.59) | 65% | (37%; 93%) | 415 |
| Sao Tome and Principe | Sub-Saharan Africa | 0.2 | 3.86 | (3.52; 4.20) | 81% | (53%; 100%) | 2520 |
| Saudi Arabia | Middle East & North Africa | 1.35 | 2.46 | (2.12; 2.80) | 100% | (72%; 100%) | 739302 |
| Senegal | Sub-Saharan Africa | 1.16 | 4.92 | (4.58; 5.26) | 100% | (72%; 100%) | 243789 |
| Serbia | Northern & Western Europe | -0.95 | 2.67 | (2.33; 3.01) | 48% | (20%; 76%) | 86006 |
| Seychelles | Sub-Saharan Africa | -0.37 | 2.10 | (1.76; 2.44) | 65% | (37%; 93%) | 1473 |
| Sierra Leone | Sub-Saharan Africa | 0.77 | 5.29 | (4.95; 5.63) | 97% | (69%; 100%) | 113293 |
| Singapore | East/South Asia & Pacific | 0.96 | 1.50 | (1.16; 1.84) | 100% | (72%; 100%) | 145939 |
| Slovakia | Northern & Western Europe | -0.76 | 1.99 | (1.65; 2.33) | 54% | (26%; 82%) | 76996 |
| Slovenia | Northern & Western Europe | -1.33 | 1.63 | (1.29; 1.97) | 38% | (10%; 66%) | 20846 |
| Solomon Islands | Oceania | 0.96 | 4.53 | (4.19; 4.87) | 100% | (72%; 100%) | 10151 |
| Somalia | Sub-Saharan Africa | 1.54 | 4.64 | (4.30; 4.98) | 100% | (72%; 100%) | 206740 |
| South Africa | Sub-Saharan Africa | -1.71 | 3.06 | (2.72; 3.40) | 27% | (0%; 55%) | 312334 |
| South Korea | East/South Asia & Pacific | -0.57 | 1.63 | (1.29; 1.97) | 59% | (31%; 87%) | 582193 |
| South Sudan | Sub-Saharan Africa | 0.96 | 5.95 | (5.61; 6.29) | 100% | (72%; 100%) | 196529 |
| Spain | Northern & Western Europe | -1.71 | 1.44 | (1.10; 1.78) | 27% | (0%; 55%) | 334167 |
| Sri Lanka | East/South Asia & Pacific | 0.77 | 2.40 | (2.06; 2.74) | 97% | (69%; 100%) | 467595 |
| Sudan | Sub-Saharan Africa | 1.16 | 4.85 | (4.51; 5.19) | 100% | (72%; 100%) | 643559 |
| Suriname | Latin America & Caribbean | -0.18 | 2.42 | (2.08; 2.76) | 70% | (42%; 98%) | 8422 |
| Swaziland | Sub-Saharan Africa | 0.87 | 4.29 | (3.95; 4.63) | 100% | (72%; 100%) | 23362 |
| Sweden | Northern & Western Europe | -2.1 | 0.99 | (0.65; 1.33) | 16% | (0%; 44%) | 41037 |
| Switzerland | Northern & Western Europe | -0.57 | 1.29 | (0.95; 1.63) | 59% | (31%; 87%) | 131422 |
| Syria | Middle East & North Africa | 1.54 | 4.55 | (4.21; 4.89) | 100% | (72%; 100%) | 310033 |
| Tajikistan | Central Asia | 0.01 | 3.67 | (3.33; 4.01) | 75% | (47%; 100%) | 121866 |
| Tanzania | Sub-Saharan Africa | 1.16 | 4.51 | (4.17; 4.85) | 100% | (72%; 100%) | 839882 |
| Thailand | East/South Asia & Pacific | -0.57 | 2.32 | (1.98; 2.66) | 59% | (31%; 87%) | 1018040 |
| Macedonia | Northern & Western Europe | -0.18 | 3.28 | (2.94; 3.62) | 70% | (42%; 98%) | 28053 |
| Timor-Leste | East/South Asia & Pacific | -0.47 | 3.43 | (3.09; 3.77) | 62% | (34%; 90%) | 11735 |
| Togo | Sub-Saharan Africa | 0.77 | 4.81 | (4.47; 5.15) | 97% | (69%; 100%) | 119706 |
| Tonga | Oceania | 0.77 | 2.32 | (1.98; 2.66) | 97% | (69%; 100%) | 1837 |
| Trinidad and Tobago | Latin America & Caribbean | 0.77 | 2.38 | (2.04; 2.72) | 97% | (69%; 100%) | 31963 |
| Tunisia | Middle East & North Africa | 1.35 | 3.60 | (3.26; 3.94) | 100% | (72%; 100%) | 262253 |
| Turkey | Middle East & North Africa | 0.01 | 2.43 | (2.09; 2.77) | 75% | (47%; 100%) | 1329187 |
| Turkmenistan | Central Asia | 0.96 | 2.98 | (2.64; 3.32) | 100% | (72%; 100%) | 115801 |
| Tuvalu | Oceania | 0.96 | 3.28 | (2.94; 3.62) | 100% | (72%; 100%) | 214 |
| Uganda | Sub-Saharan Africa | 0.96 | 4.87 | (4.53; 5.21) | 100% | (72%; 100%) | 579451 |
| Ukraine | Eastern Europe | -0.18 | 2.98 | (2.64; 3.32) | 70% | (42%; 98%) | 825827 |
| United Arab Emirates | Middle East & North Africa | 1.35 | 2.13 | (1.79; 2.47) | 100% | (72%; 100%) | 256650 |
| UK | Northern & Western Europe | -1.9 | 1.04 | (0.70; 1.38) | 21% | (0%; 49%) | 350291 |
| USA | North America | -1.33 | 1.02 | (0.68; 1.36) | 38% | (10%; 66%) | 3033797 |
| Uruguay | Latin America & Caribbean | -1.9 | 1.57 | (1.23; 1.91) | 21% | (0%; 49%) | 17105 |
| Uzbekistan | Central Asia | 0.96 | 3.76 | (3.42; 4.10) | 100% | (72%; 100%) | 679721 |
| Vanuatu | Oceania | 0.2 | 3.70 | (3.36; 4.04) | 81% | (53%; 100%) | 4012 |
| Venezuela | Latin America & Caribbean | -0.57 | 1.88 | (1.54; 2.22) | 59% | (31%; 87%) | 395373 |
| Viet Nam | East/South Asia & Pacific | 0.2 | 3.33 | (2.99; 3.67) | 81% | (53%; 100%) | 1784102 |
| West Bank | Middle East & North Africa | -0.09 | 3.16 | (2.82; 3.50) | 73% | (45%; 100%) | 55820 |
| Yemen | Middle East & North Africa | 1.16 | 4.56 | (4.22; 4.90) | 100% | (72%; 100%) | 457806 |
| Zambia | Sub-Saharan Africa | 0.96 | 4.02 | (3.68; 4.36) | 100% | (72%; 100%) | 247049 |
| Zimbabwe | Sub-Saharan Africa | 0.77 | 4.56 | (4.22; 4.90) | 97% | (69%; 100%) | 254897 |
|  | | | | | | | |
